# Improving awareness, diagnosis and management of invasive fungal infections in Ghana: establishment of the Ghana Medical Mycology Society

**DOI:** 10.1093/mmy/myac069

**Published:** 2022-09-08

**Authors:** Bright K Ocansey, Edmund A Dadzie, Stephen K Eduful, Martin Agyei, Mary-Magdalene Osei, Peter Puplampu, Isabella Asamoah, Rita O Oladele, Iriagbonse I Osaigbovo, Jane S Afriyie-Mensah, Japheth A Opintan, Samuel Essien-Baidoo, Arunaloke Chakrabarti, Martin Hoenigl, David W Denning, Malcolm D Richardson

**Affiliations:** Division of Evolution, Infection and Genomics, Faculty of Biology, Medicine and Health, University of Manchester, Manchester M13 9NT, UK; Department of Health and Allied Sciences, Baldwin University College, Accra GA-082, Ghana; Department of Medical Laboratory Sciences, School of Biomedical and Allied Health Sciences, University of Ghana, Accra GA-337, Ghana; Department of Medicine, School of Medical Sciences, Kwame Nkrumah University of Science and Technology, Kumasi AK-448, Ghana; Department of Medical Microbiology, University of Ghana Medical School, Accra GA-270, Ghana; Department of Medicine and Therapeutics, University of Ghana Medical School, Accra GA-221, Ghana; Department of Medicine and Therapeutics, Korle-Bu Teaching Hospital, Accra GA-221, Ghana; Department of Medicine and Therapeutics, Korle-Bu Teaching Hospital, Accra GA-221, Ghana; Department of Medical Microbiology, College of Medicine, University of Lagos, Lagos 101017, Nigeria; Department of Medical Microbiology, College of Medical Sciences, University of Benin, Benin City 300213, Nigeria; Department of Medicine and Therapeutics, University of Ghana Medical School, Accra GA-221, Ghana; Department of Medicine and Therapeutics, Korle-Bu Teaching Hospital, Accra GA-221, Ghana; Department of Medical Microbiology, University of Ghana Medical School, Accra GA-270, Ghana; Department of Medical Laboratory Sciences, School of Allied Health Sciences, University of Cape Coast, Cape Coast CC-071, Ghana; Department of Medical Microbiology, Postgraduate Institute of Medical Education and Research, Chandigarh 160012, India; Section of Infectious Diseases and Tropical Medicine, Division of Pulmonology, Medical University of Graz, Graz 8036, Austria; Division of Infectious Diseases and Global Public Health, University of California San Diego, San Diego, CA 92093-0021, USA; Division of Evolution, Infection and Genomics, Faculty of Biology, Medicine and Health, University of Manchester, Manchester M13 9NT, UK; Division of Evolution, Infection and Genomics, Faculty of Biology, Medicine and Health, University of Manchester, Manchester M13 9NT, UK; Mycology Reference Centre, Wythenshawe Hospital, Manchester University NHS Foundation Trust, Manchester M23 9LT, UK

**Keywords:** Ghana, invasive fungal infections, diagnostics, antifungal agents, research

## Abstract

Invasive fungal infections (IFIs) and medical mycology receive little attention in Ghana. However, the present evolution of biomarker assays for IFIs, offers an opportunity for an increased access to fungal laboratory testing in resource-limited settings, and probably make a case for availability of essential antifungal agents. Using surveys and personal communications, the state of medical mycology and IFI in Ghana were highlighted. Inadequate awareness and insufficient access to fungal diagnostics and therapeutics were identified as the key challenges, the establishment of the Ghana Medical Mycology Society was discussed, and recommendations were made to improve the status quo.

## Introduction

Globally, the burden of invasive fungal infections (IFIs) continue to increase due to the expansion of the population at risk and emerging risk groups including the coronavirus disease 2019 (COVID-19).^1^^,^^2^ Recent concerns are, increasing antifungal resistance, multi-drug-resistant *Candida auris* and emergence of rare infections.^3^^–^^5^ Notwithstanding, IFIs receive little attention in many parts of the world, particularly Africa that is disproportionately affected by IFIs due to the HIV and tuberculosis epidemic and the tropical climate.^6^ The Leading International Fungal Education (LIFE) and Global Action for Fungal Infections (GAFFI) burden-estimate projects have stimulated interest in medical mycology in many African countries including Ghana. The evolution of rapid and simple fungal biomarker assays and access programs for essential antifungal agents offers a unique opportunity for low- and middle-income countries (LMICs) to improve diagnosis and management of patients suffering from IFIs. In this report, we highlight the current state of IFIs and medical mycology in Ghana, by identifying the key challenges and discuss the formation of the Ghana Medical Mycology Society (GMMS), formerly Ghana Medical Mycology Group (GMMG) and recommendations to improve the status quo.

## Methods

Information was gathered from a previously published study on IFIs in Ghana,^7^ findings from three unpublished gap analysis surveys to evaluate IFI awareness among health sciences students, availability and accessibility to fungal laboratory tests and essential antifungal agents in Ghana, and personal communications with relevant stakeholders were used to prepare this report.

## Results and discussion

In 2019, about 4% of the Ghanaian population was estimated to suffer from serious fungal infections yearly, including over 35 000 affected by IFIs.^7^ This estimate may not represent a true reflection of a burden of IFIs in Ghana, because some of the prevalence or incidence rates used were borrowed from regional and global studies due to absence of local epidemiological data. The study also reported that, data on IFIs generally consist of few sporadic case reports where diagnoses were mostly made post-mortem, probably indicating a low index of suspicion.^7^ Furthermore, in some of the case reports, the authors bemoaned the insufficient clinical suspicion and inaccessibility to critical fungal laboratory tests and essential antifungal agents. Real life epidemiological studies are needed for a true burden to be appreciated. For example, a recently published study revealed that disseminated histoplasmosis may be more common than cryptococcal meningitis in Ghana contradicting the findings from the model study.^8^ The few epidemiological studies are often spearheaded by foreign clinicians and the upskilled diagnostic capacity declines after the study.^9^^,^^10^ Going forward, study investigators must engage policy makers and clinical leads towards drawing a framework for implementation and ensuring the integration of the research laboratory testing into clinical practice.

There are currently no surveillance or case notification programs for any IFI in Ghana. Meanwhile, the steady prevalence rate (∼2.0%) of HIV, TB burden (286/100 000), increase in cancer rates (2000–5000 increase per year in the last decade), gradual introduction of kidney (over 20 live donor transplants in the last decade) and stem cell transplant procedures and expanding ICU beds (149 beds, over 100% increase since the COVID-19 pandemic) also makes way for the significant occurrence of IFIs.^11^^–^^15^ Unpublished data from three gap analysis surveys in Ghana, suggest that awareness on IFIs among final year health sciences students was inadequate and the availability of fungal diagnostics and antifungal drugs (Table 1) is poor, particularly in relation to the WHO recommendations.^16–19^

**Table 1.** Availability of fungal diagnostics and essential antifungal agents in Ghana.

Test	Availability	On Ghana EDL	On WHO EDL^16^^,^^17^
Direct microscopy (KOH)	Often	N/A	Yes
Histopathology (fungal stains)	Occasionally	N/A	Yes
Fungal culture	Rarely	N/A	Yes
Immunoassay	Rarely	N/A	Yes (*Aspergillus* Ag and Ab, CrAg, *Histoplasma* GM)
Molecular	Rarely	N/A	*Pneumocystis jirovecii* PCR
Antifungal susceptibility	Rarely	N/A	Yes and WHO GLASS programme (*Candida* spp.)
			
**Antifungal agent**	**Availability**	**On Ghana EML^18^**	**On WHO EML^19^**
			
Fluconazole	Often	Yes	Yes
Itraconazole	Occasionally	Yes	Yes
Amphotericin B	Rarely	No	Yes
Flucytosine	Rarely	No	Yes
Voriconazole	Rarely	No	Yes
Natamycin	Rarely	No	Yes

EDL-essential diagnostics list, N/A-not applicable (because Ghana has no EDL currently), KOH-Potassium hydroxide, Ag-antigen, Ab-antibody, CrAg- Cryptococcal antigen, GM- Galactomannan, PCR-polymerase chain reaction, GLASS-Global Antimicrobial Resistance Surveillance System, EML- essential medicines list.

In Ghana, medical mycology is underdeveloped and attracts little interest in academia and professional practice. Teaching hours allocated for mycology are limited and options for specialization in mycology are almost non-existent. Moreover, numbers of educators, researchers, and trainers are also woefully inadequate. Unlike, plant mycology, there are presently no established medical mycology units/divisions in health/medical institutions in Ghana.

To increase awareness, a non-governmental organization, Fungal Infections Kare Initiative (FIKI) Ghana was founded in 2019 led by BKO and EAD. FIKI Ghana organized two sensitization seminars with the second inaugurating the GMMG. The foundation also facilitated the three gap analysis surveys mentioned previously with financial support from the Fungal Infection Trust (FIT). FIKI Ghana also established collaborative links with the International Society for Human and Animal Mycology (ISHAM), Centre for Disease Control (CDC) Mycotic Disease Branch, UK National Aspergillosis Centre, and Mycology Reference Centre, Manchester that have been extended to GMMS and continue to strengthen it. The role of professional networks in the advancement of unpopular fields such as medical mycology cannot be overemphasized and strongly demonstrated by the Medical Mycology Society of Nigeria (MMSN) that charted a model on how societies can contribute to combating IFIs in LMICs.^20^

GMMS, is a network of multidisciplinary cadre of healthcare professionals (HCP) mainly clinicians and scientists as well as health science researchers and academics with interest in medical mycology or fungal infections. The current membership includes infectious disease specialists/consultants, dermatologists, clinical microbiologists, medical laboratory scientists, pulmonologists, and pharmacists.

GMMS has a vision to reduce illness and death associated with fungal diseases in Ghana. The GMMS’ mission is to increase awareness, build diagnostic and therapeutic capacity, campaign for access to critical diagnostics and antifungal agents, drive research, and influence the gradual integration of the discipline into health systems in Ghana, mainly through an eight-itemized activity schedule (Table 2). The society also circulates advocacy leaflets and disseminates links of free training, educational, and clinical resources. Since its inception in February 2020, GMMS has successfully organized several meetings and activities in three consecutive years. The society has also occasionally served as a platform to provide guidance on diagnosis and treatment of IFIs to Ghanaian clinicians and scientists with support from our international affiliations. GMMS continuously shares participation details of meetings in the field of medical mycology to members and urges to disseminate to colleagues. Members are constantly encouraged to register with ISHAM, to take advantage of associated benefits and currently have 20% of our members registered with ISHAM.

**Table 2.** GMMS activities.

Activity	Frequency
Journal club	Quarterly
Case report forum	Quarterly
Serious mycoses symposium	Annual (during fungal diseases awareness week)
Aspergillosis lecture	Annual (on World Aspergillosis Day)
Training workshop	Annual
National scientific conference	Biennial
Fungal infection talk (facility based)	Bi-annual
Question of the week (via WhatsApp)	Weekly

The recommended framework for a short term, is for the government to adopt and upgrade the WHO Essential Diagnostic and Medicine lists respectively, particularly in the tertiary hospitals. Also, training of technical laboratory staff and clinicians in these institutions will lay a solid foundation for the integration of clinical mycology in the healthcare system broadly. From a practical perspective, the easiest tests to adopt are the lateral flow assays, especially cryptococcal and *Histoplasma* antigen and *Aspergillus* antibody in HIV and TB control programmes, respectively. From this start, direct microscopy and fungal culture could be the next step. From a public health and clinical perspective, undertaking a surveillance of key fungal diseases through a hospital and laboratory reporting network, would be valuable in improving local epidemiology and encouraging and attracting clinical research with international funding.

## Conclusion

Ghana is challenged with the underrecognition of IFIs and underdevelopment of medical mycology. In face of the numerous challenges, the GMMS remains resolute and optimistic to act as a major driver to increase awareness, advance the diagnostic and therapeutic skills of clinicians and scientists, improve access to fungal diagnostics and antifungal drugs, and champion the integration medical mycology into health systems in Ghana.
